# Monocyte distribution width (MDW) and DECAF: two simple tools to determine the prognosis of severe COPD exacerbation

**DOI:** 10.1007/s11739-024-03632-5

**Published:** 2024-05-09

**Authors:** Carlos A. Amado, Cristina Ghadban, Adriana Manrique, Joy Selene Osorio, Milagros Ruiz de Infante, Rodrigo Perea, Laura Gónzalez-Ramos, Sergio García-Martín, Lucia Huidobro, Javier Zuazaga, Patricia Druet, Pedro Argos, Claudia Poo, Ma Josefa Muruzábal, Helena España, Guido Andretta

**Affiliations:** 1 Department of Pulmonology, Hospital Universitario Marqués de Valdecilla, Av Valdecilla SN, 39005 Santander, Spain; 2 University of Cantabria, Santander, Spain; 3 IDIVAL (Instituto de Investigación Biomédica de Cantabria), Santander, Spain; 4 Department of Anesthesiology, Hospital Sierrallana, Torrelavega, Spain; 5 Department of Pulmonology, Hospital de Laredo, Laredo, Spain; 6 Department of Hematology, Hospital Universitario Marqués de Valdecilla, Santander, Spain

**Keywords:** COPD, MDW, DECAF, Exacerbation, Prognosis

## Abstract

Monocyte distribution width (MDW) has been associated with inflammation and poor prognosis in various acute diseases. Chronic obstructive pulmonary disease (COPD) exacerbations (ECOPD) are associated with mortality. The objective of this study was to evaluate the utility of the MDW as a predictor of ECOPD prognosis. This retrospective study included patient admissions for ECOPD. Demographic, clinical and biochemical information; intensive care unit (ICU) admissions; and mortality during admission were recorded. A total of 474 admissions were included. MDW was positively correlated with the DECAF score (*r* = 0.184, *p* < 0.001) and C-reactive protein (mg/dL) (*r* = 0.571, *p* < 0.001), and positively associated with C-RP (OR 1.115 95% CI 1.076–1.155, *p* < 0.001), death (OR 9.831 95% CI 2.981– 32.417, *p* < 0.001) and ICU admission (OR 11.204 95% CI 3.173–39.562, *p* < 0.001). High MDW values were independent risk factors for mortality (HR 3.647, CI 95% 1.313–10.136, *p* = 0.013), ICU admission (HR 2.550, CI 95% 1.131–5.753, *p* = 0.024), or either mortality or ICU admission (HR 3.084, CI 95% 1.624–5.858, *p* = 0.001). In ROC analysis, a combined MDW–DECAF score had better diagnostic power (AUC 0.777 95% IC 0.708–0.845, *p* < 0.001) than DECAF (*p* = 0.023), MDW (*p* = 0.026) or C-RP (*p* = 0.002) alone. MDW is associated with ECOPD severity and predicts mortality and ICU admission with a diagnostic accuracy similar to that of DECAF and C-RP. The MDW– DECAF score has better diagnostic accuracy than MDW or DECAF alone in identifying mortality or ICU admission.

**Supplementary Information:**

The online version contains supplementary material available at 10.1007/s11739-024-03632-5.

## Introduction

Chronic obstructive pulmonary disease (COPD) is a leading cause of morbidity and mortality worldwide [1]. COPD exacerbation (ECOPD) is a major outcome of this disease and ECOPD severity is associated with future mortality risk [2], a decline in FEV1 [3], diminished quality of life [4] and future ECOPD [5, 6]. Moreover, ECOPD hospitalizations are associated with high mortality rates [7, 8]. Nonetheless, few tools are available to help clinicians to determine ECOPD prognosis in hospitalized patients. These tools include C-reactive protein (C-RP), the only biomarker recommended by GOLD [9] to evaluate ECOPD severity, and the dyspnea–eosinopenia–consolidation–acidemia–atrial fibrillation (DECAF) prognostic score, which uses several clinical variables (dyspnea, eosinopenia, consolidation, acidemia and atrial fibrillation) to establish the prognosis of severe ECOPD [10]. Therefore, new parameters must be investigated in clinical practice to determine prognosis in patients with severe ECOPD.

The inflammatory response during ECOPD is mediated by the activation of neutrophilic and lymphocytic inflammation [11]. Circulating neutrophils and monocytes are involved in the initial response to pathogenic organisms that cause ECOPD [12]. Monocyte distribution width (MDW) reflects the degree of change in circulating monocyte volume (“heterogeneity”) in response to proinflammatory signals elicited by infectious organisms. MDW determination is easily automated, inexpensive and quickly obtained as part of routine blood counts. Recent data have suggested that an increase in MDW may be useful in early detection of all-cause sepsis [13–21]. However, few studies have been performed in acute respiratory conditions; one example is a study in a limited number of patients with COVID-19 [22–24]. Some studies have suggested that MDW may be associated with the neutrophil/lymphocyte ratio, a biomarker associated with ECOPD prognosis [11].

We hypothesized that high MDW at hospital admission might serve as a prognostic inflammatory biomarker in patients with ECOPD and that combining the MDW with the DECAF score might further increase the prognostic value. To our knowledge, this aspect has not previously been studied in this setting.

## Methods

This was a multicenter observational retrospective study in which clinical records from patients admitted for ECOPD between March 1st, 2020, and March 1st, 2023, to three public hospitals of the Servicio Cántabro de Salud network in the Cantabria community in northern Spain were reviewed. The ethics committee of our institution (2023.061) approved the study.

### Participants

We recruited patients hospitalized for ECOPD between March 2020 and March 2023.

The inclusion criteria were as follows: (1) Patients older than 40 years previously diagnosed with COPD according to the GesEPOC guidelines [25] and (2) Patients hospitalized because of ECOPD. ECOPD was defined by an increase in more than one of the following respiratory symptoms: dyspnea, sputum purulence, increased sputum, cough or wheezing; symptoms persisting for at least 2 consecutive days; and symptoms requiring treatment with antibiotics and/or systemic steroids [9].

The exclusion criteria were as follows: (1) Patients with a diagnosis other than ECOPD at discharge; (2) Patients without clinical, biochemical or microbiological data consistent with ECOPD; (3) Patients with active cancer, leukemia, lymphoma, lymphoproliferative disorders, bone marrow diseases or AIDS; (4) Patients with vitamin B12 or folic acid deficiency; and (5) Patients treated with immunosuppressants (including systemic corticosteroids) or drugs causing macrocytosis.

### Measurements

Stable spirometry was performed according to the Spanish Society of Pulmonology and Thoracic Surgery (SEPAR) protocol [26] less than 1 year before admission. Patient age; sex; smoking status; number of moderate and severe ECOPD events 1 year before admission; comorbidities included in the Charlson index [27]; basal mMRC dyspnea score; and dates of hospital admission, ICU admission and/or death during hospitalization were retrospectively recorded. The DECAF score [25] was calculated for all patients. The MDW and results of routine blood tests performed after arrival at the emergency department of each center were retrospectively collected.

Routine hematological and biochemical analytes were measured with automated assays. Specifically, C-RP was measured with Siemens traceable enzymatic method assays (Atellica Analyzer, Siemens, Germany).

MDW was measured in the emergency laboratory of each center with the same DxH 900 analyzer (Beckman Coulter. Inc., Brea, California, USA), according to the manufacturer´s instructions.

### Statistical analysis

Data are presented as mean ± SD for normally distributed data or median (interquartile range) for nonparametric data. We calculated sample sizes in *Stata Statistical Software: Release 15*. College Station, TX: StataCorp LLC.), with an α level of 0.05 and a β level of 0.2. Differences between groups were analyzed with unpaired *t* tests for parametric data or Mann–Whitney tests for nonparametric data. Normality of distribution was evaluated with the Kolmogorov–Smirnov test. We evaluated the correlation between MDW and other variables with Spearman tests. Evaluation of MDW as a dichotomized variable was established with a cutoff at 21.5 units, according to previous studies [28–30] performed in the same setting and using similar laboratory protocols as our study. We evaluated cross-sectional associations with high versus low MDW through univariate and multivariate logistic regression, with outcome variables of mortality; ICU admission; and a composite end point including in-hospital all-cause mortality and escalation to ICU admission. We used Kaplan–Meier estimates to calculate the proportion of participants experiencing mortality; ICU admission; and a composite end point including in-hospital all-cause mortality and escalation to ICU admission due to ECOPD over time. We performed univariate and multivariate Cox proportional risk analysis in SPSS version 25.00. A receiver operating characteristic (ROC) curve and the area under the ROC curve (AUC) were used to assess the diagnostic value of MDW for a composite end point including in-hospital all-cause mortality and escalation to ICU admission as the outcome variables. ROC curve analysis was performed in MEDCALC version 11.6.1.0 (MedCalc Software, Mariakerke, Belgium). Differences with *p* values < 0.05 were considered significant. All reported *p* values were two sided.

## Results

### Patient characteristics

**Fig. 1:**
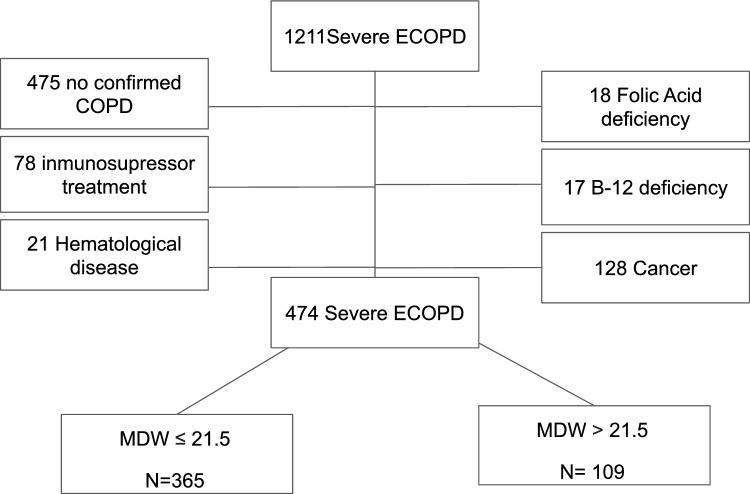
Flowchart for patient selection

A total of 474 pw ECOPD were ultimately included in the study (flowchart for patient selection in Fig. 1; demographic, clinical and biochemical data in Table 1). The median patient age was 75 (67–82) years, and most patients were men (67.7%). The prevalence of current smokers (32.9%) was high. Most patients had a previous admission for ECOPD [178 (37.6%)] and moderate or severe obstruction, on the basis of FEV1 (%) 50 (35–67). A total of 26 patients died (5.5%); although ICU admission was not frequent [31 (6.5)], a composite end point including mortality or ICU admission reached 11% (52 patients). The blood MDW levels were 19.2 (17.2–21.2) units, the blood leucocyte levels were 9900 (7300–13525) cells/µL and the serum C-RP levels were 4.4 (1.5–11.7) mg/dL.

**Table 1:** Demographic, clinical and biochemical characteristics of all patients and patients with MDW ≤ 21.5 units vs MDW > 21.5 units

Variable	Total (*n* = 474)	MDW ≤ 21.5 (*n* = 365)	MDW > 21.5 (*n* = 109)	p*
Age (years)	75 (67–82)	74(67–82)	76(66–84)	0.338
Sex male *n* (%)	321(67.7)	248(67.9)	73(67)	0.849
Current smokers *n* (%)	156 (32,9)	127(34.8)	29(26.6)	0.110
Charlson index	2(1–4)	2(1–4)	2(2–4)	0.742
Previous admission *n* (%)	178(37.6)	135(37.1)	43(39.4)	0.655
2 or more exacerbations during the previous year *n* (%)	151(31.9)	117(32.1)	34(31.2)	0.865
Basal mMRC score 0/I/II/III/IV n (%)	35/116/137/139/47	27(7.4)/84(23)/108(29.6)/108(29.6)/38(10.4)	8(7.3)/32(29.4)/29(26.6)/31(28.4)/9(8.3)	0.723
**DECAF score**	**2(1–3)**	**2(1–2.5)**	**2(2–3)**	** < 0.001**
**Mortality during hospitalization *n* (%)**	**26(5.5)**	**8(2.2)**	**18(16.5)**	** < 0.001**
**ICU admission during hospitalization *n* (%)**	**31(6.5)**	**14(3.8)**	**17(15.6)**	** < 0.001**
**Composite end point (mortality or ICU admission) *n* (%)**	**52(11)**	**22(6)**	**30(27)**	** < 0.001**
BMI (kg/m^2^)	28(24–32)	28(24–32)	28(23–32)	0.448
FVC (L)	2.45 ± 0.86	2.43 ± 0.83	2.529 ± 0.99	0.155
FVC (% predicted)	78(65–93)	78(64–92)	79(70–95)	0.119
**FEV**_**1**_** (L)**	**1.10(0.84–1.63)**	**1.09(0.82–1.55)**	**1.23(0.92–1.73)**	**0.018**
**FEV**_**1**_** (% predicted)**	**50(35–67)**	**50(33–64)**	**57(43–69)**	**0.004**
**FEV**_**1**_**/FVC**	**50(41–59)**	**49(40–59)**	**54(43–60)**	**0.04**
**MDW (units)**	**19.2(17.2–21.2)**	**18.3(16.7–19.8)**	**23.4(22.4–24.6)**	** < 0.001**
**C-reactive protein (mg/dl)**	**4.4(1.5–11.7)**	**2.9(1–7.9)**	**12.6(5.0–20.8)**	** < 0.001**
pH	7.40(7.36–7.42)	7.40(7.36–7.41)	7.40(7.35–7.43)	0.414
paO_2_ (mmHg)	67(58.5–82)	68(58–82)	63(58–82)	0.159
paCO_2_ (mmHg)	44(36–54)	43(36–55)	44(38–49)	0.587
HCO_3_ (mmol/L)	27(22–31)	27(22–31)	27(23–29)	0.817
Leucocytes (cells/µL)	9900(7300–13525)	10,000(7500–13500)	9500(6800–14350)	0.828
Neutrophils (cells/µL)	7700(5400–11025)	7700(5500–10750)	7400(5000–11500)	0.773
Monocytes (cells/µL)	800(500–1100)	800(500–1100)	800(500–1200)	0.945
**Lymphocytes (cells/µL)**	**1000(600–1600)**	**1000(600–1600)**	**800(500–1400)**	**0.049**
**Basophils (cells/µL)**	**0(0–100)**	**0(0–100)**	**0(0–50)**	**0.015**
**Eosinophils (cells/µL)**	**100(0–125)**	**100(0–200)**	**0(0–100)**	** < 0.001**
**Netrophils/lymphocytes**	**7.23(4.68–14.00)**	**6.91(4.58–12.18)**	**9.60(5.20–17.83)**	**0.008**

Bold font indicates statistical significance

*MDW* monocyte distribution width, *mMRC *modified Medical Research Council dyspnea score, *FVC *forced vital capacity, *FEV1* forced expiratory volume in the first second, *DECAF *dyspnea, eosinopenia, consolidation, acidemia and atrial fibrillation score, *BMI* body mass index

*p value for patients with MDW ≤ 21.5 vs upper MDW

The group of patients with ECOPD and high MDW tended to have high ECOPD severity, on the basis of the DECAF score, mortality, ICU admission rate and elevated inflammatory parameters, such as C-RP and the neutrophil to lymphocyte ratio. Notably, the group with high MDW had slightly higher FEV1 values. However, the two groups had similar age, sex distribution, smoking habits, basal mMRC scores and ECOPD history.

### MDW correlations

MDW was positively correlated with the DECAF score (*r* = 0.184, *p* < 0.001), FEV1 (L) (*r* = 0.153, *p* = 0.001), FEV1 (% predicted) (*r* = 0.149, *p* = 0.001), FEV1/FVC (*r* = 0.106, *p* = 0.022), C-RP (mg/dL) (*r* = 0.571, *p* < 0.001) and neutrophil to lymphocyte ratio (*r* = 0.135, *p* = 0.003).

MDW was negatively correlated with basophil count (*r* = −0.111, *p* = 0.016) and eosinophil count (*r* = −0.288, *p* < 0.001).

MDW did not correlate with age (*r* = 0.025, *p* = 0.588), Charlson score (*r* = 0.056, *p* = 0.291), BMI (*r* = −0.02, *p* = 0.978), FVC (L) (*r* = 0.069, *p* = 0.145), FVC FEV1 (% predicted) (*r* = 0.077, *p* = 0.099), paO_2_ (*r* = -0.084, *p* = 0.069), paCO_2_ (*r* = −0.088, *p* = 0.056), HCO_3_ (*r* = −0.065, *p* = 0.173), pH (*r* = 0.088, *p* = 0.056), leucocyte count (*r* = 0.024, *p* = 0.597), neutrophil count (*r* = 0.063, *p* = 0.172), monocyte count (*r* = 0.050, *p* = 0.274) or lymphocyte count (*r* = −0.080, *p* = 0.081).

### Association of MDW with ECOPD characteristics

Table 2 highlights the associations of MDW with baseline ECOPD characteristics. Multivariate logistic regression analysis showed that high MDW was positively associated with C-RP (OR 1.115 95% CI 1.076–1.155, *p* < 0.001), death (OR 9.831 95% CI 2.981–32.417, *p* < 0.001) and ICU admission (OR 11.204 95% CI 3.173–39.562, *p* < 0.001); however, no associations with other ECOPD characteristics were observed.

**Table 2:** Univariate and multivariate logistic regression analysis of the associations between ECOPD characteristics and high MDW

		MDW > 21.5 units			
		Univariate		Multivariate	
		OR (95% CI)	*p*	OR (95% CI)	*p
Age (years)		1.009 (0.988–1.030)	0.880	1.007 (0.976–1.039)	0.657
Sex					
	Male	1		1	
	Female	0.957 (0.607–1.509)	0.185	0.880(0.477–1.623)	0.682
Smoking status					
	Former smoker	1		1	
	Current smoker	0.679 (0.422–1.094)	0.112	1.228 (0.608–2.480)	0.566
Previous exacerbations					
	0–1	1		1	
	≥ 2	0.961 (0.606–1.524)	0.865	1.163 (0.566–2.389)	0.682
Previous hospitalization					
	0	1		1	
	≥ 1	1.105 (0.712–1.714)	0.655	0.728 (0.370–1.431)	0.357
Basal mMRC		0.909 (0.749–1.103)	0.333	0.931 (0.706–1.229)	0.614
Charlson		0.993 (0.878–1.124)	0.915	0.997 (0.851–1.169)	0.972
FEV1 (%)		1.001 (0.999–1.003)	0.324	1.002 (0.999–1.004)	0.145
**C-RP (mg/dL)**		**1.120 (1.089–1.151)**	** < 0.001**	**1.115 (1.076–1.155)**	** < 0.001**
DECAF score		**1.518 (1.249–1.845)**	**0.001**	1.172 (0.905–1.517)	0.230
Leucocytes (cells/microL)		1.000 (1.000–1.000)	0.826	1.000 (0.999–1.000)	0.437
Neutrophils (cells/microL)		1.000 (1.000–1.000)	0.164	1.000 (1.000–1.001)	0.495
Monocytes (cells/microL)		1.000 (1.000–1.000)	0.947	1.000 (0.999–1.001)	0.743
Lymphocytes (cells/microL)		1.000 (1.000–1.000)	0.122	1.000 (1.000–1.001)	0.362
Basophils (cells/microL)		0.997 (0.993–1.001)	0.107	1.000 (0.995–1.005)	0.960
Eosinophils (cells/microL)		**0.997 (0.995–0.999)**	**0.002**	0.998 (0.996–1.000)	0.102
Netrophils/lymphocytes		1.000 (1.000–1.000)	0.412	1.000 (1.000–1.000)	0.373
pH		0.853 (0.040–18.117)	0.919	369.808 (0.837–163,329.086)	0.057
paCO_2_ (mmHg)		1.000 (0.986–1.013)	0.968	0.996 (0.969–1.023)	0.748
HCO_3_ (mmHg		0.998 (0.982–1.014)	0.809	1.001 (0.987–1.016)	0.852
paO_2_ (mmHg)		0.998 (0.987–1.010)	0.787	1.000 (0.986–1.015)	0.965
Death					
	Survivors	1		1	
	**Death**	**3.720 (3.720–20.944)**	** < 0.001**	**9.831 (2.981–32.417)**	** < 0.001**
ICU admittance					
	Not admitted to ICU	1		1	
	**Admitted to ICU**	**4.633 (2.202–9.746)**	** < 0.001**	**11.204 (3.173–39.562)**	** < 0.001**

Bold font indicates statistical significance

*MDW *monocyte distribution width, *mMRC *modified Medical Research Council dyspnea score, *FEV1* forced expiratory volume in the first second, *DECAF* dyspnea, eosinopenia, consolidation, acidemia and atrial fibrillation score

*p value for patients with MDW ≤ 21.5 vs upper MDW

### MDW as a predictor of mortality, ICU admission or both

Among 474 patients with ECOPD included in the study, 109 had a high MDW. Table 1 shows the clinical characteristics of both groups. A total of 26 patients died during hospitalization (18 in the high MDW group), 31 patients were admitted to the ICU (17 in the high MDW group) and 52 patients met the composite end point including mortality or ICU admission (30 in the high MDW group).

#### Predictors of mortality

**Fig. 2:**
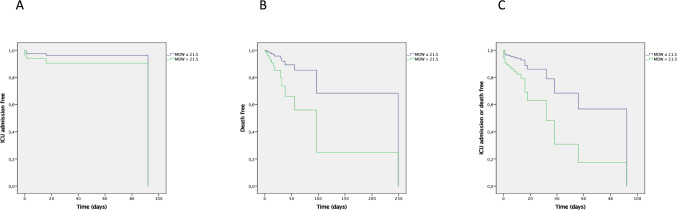
High MDW (> 21.5 units) as a predictor of **A** ICU admission, **B** mortality and **C** ICU admission or mortality. *MDW* monocyte distribution width

Univariate Cox proportional risk analysis indicated that age (*p* = 0.04), sex (women) (*p* = 0.292), MDW (*p* = 0.001) and high MDW (higher than 21.5) (*p* = 0.002), but not smoking status (*p* = 0.064), FEV1 (*p* = 0.626), Charlson index (*p* = 0.101), DECAF score (*p* = 0.069), C-RP (*p* = 0.127) or neutrophil to lymphocyte ratio (*p* = 0.416) were predictors of mortality during hospitalization due to ECOPD. Multivariate Cox proportional risk analysis revealed that the absolute values of MDW (HR 1.171, CI 95% 1.073–1.277, *p* < 0.001) (Table 3) and high MDW (HR 3.647, CI 95% 1.313–10.136, *p* = 0.013) (Table 3, Fig. 2) were independent risk factors for mortality during hospitalization for ECOPD.

**Table 3:** Cox regression analysis showing absolute and dichotomized values of MDW as a predictor of death, ICU admission and the composite end point (mortality or ICU admission)

Variable	B	Wald	*p*	HR	95% CI HR	
					Lower	Upper
**MDW (absolute value in units)**						
** Mortality**	**0.158**	**12.648**	** < 0.001**	**1.171**	**1.073**	**1.277**
** ICU admittance**	**0.083**	**4.866**	**0.027**	**1.086**	**1.009**	**1.169**
** Composite end point**	**0.115**	**18.839**	** < 0.001**	**1.122**	**1.065**	**1.182**
**Dichotomized MDW (> 21.5 units vs rest of patients)**						
** Mortality**	**1.294**	**6.158**	**0.013**	**3.647**	**1.313**	**10.136**
** ICU admittance**	**0.936**	**5.088**	**0.024**	**2.550**	**1.131**	**5.753**
** Composite end point**	**1.126**	**11.840**	**0.001**	**3.084**	**1.624**	**5.858**

Bold font indicates statistical significance

*MDW* monocyte distribution width, *DECAF *dyspnea, eosinopenia, consolidation, acidemia and atrial fibrillation score, *composite end point *mortality or ICU admission

^*^All variables adjusted by age, sex, Charlson index, forced expiratory volume in the first second, smoking status, DECAF score, C-reactive protein, neutrophil to lymphocyte ratio

#### Predictors of ICU admission

Univariate Cox proportional risk analysis indicated that age (*p* < 0.001), smoking status (*p* = 0.007), DECAF score (*p* < 0.001), C-RP (*p* = 0.002), MDW (*p* < 0.001) and high MDW (higher than 21.5) (*p* < 0.001), but not sex (*p* = 0.292), FEV1 (*p* = 0.802), Charlson index (*p* = 0.995) or neutrophil to lymphocyte ratio (*p* = 0.654) were predictors of ICU admission. Multivariate Cox proportional risk analysis revealed that absolute values of MDW (HR 1.086, CI 95% 1.009–1.169, *p* = 0.027) (Table 3) and high MDW (HR 2.550, CI 95% 1.131–5.753, *p* = 0.024) (Table 3, Fig. 2) were independent risk factors for ICU admission.

#### Predictors of the composite end point (mortality or ICU admission)

Univariate Cox proportional risk analysis indicated that age (*p* = 0.047), DECAF score (*p* < 0.001), C-RP (*p* < 0.001), MDW (*p* < 0.001) and high MDW (higher than 21.5) (*p* < 0.001), but not smoking status (*p* = 0.072), sex (*p* = 0.767), FEV1 (*p* = 0.786), Charlson index (*p* = 0.100) or neutrophil to lymphocyte ratio (*p* = 0.754) were predictors of the composite end point (mortality or ICU admission). Multivariate Cox proportional risk analysis revealed that absolute values of MDW (HR 1.122, CI 95% 1.065–1.182, *p* < 0.001) (Table 3) and high MDW (HR 3.084, CI 95% 1.624–5.858, *p* = 0.001) (Table 3, Fig. 2) were independent risk factors for the composite end point (mortality or ICU admission).

### Potential utility of the MDW and DECAF–MDW score in predicting the composite end point

**Fig. 3:**
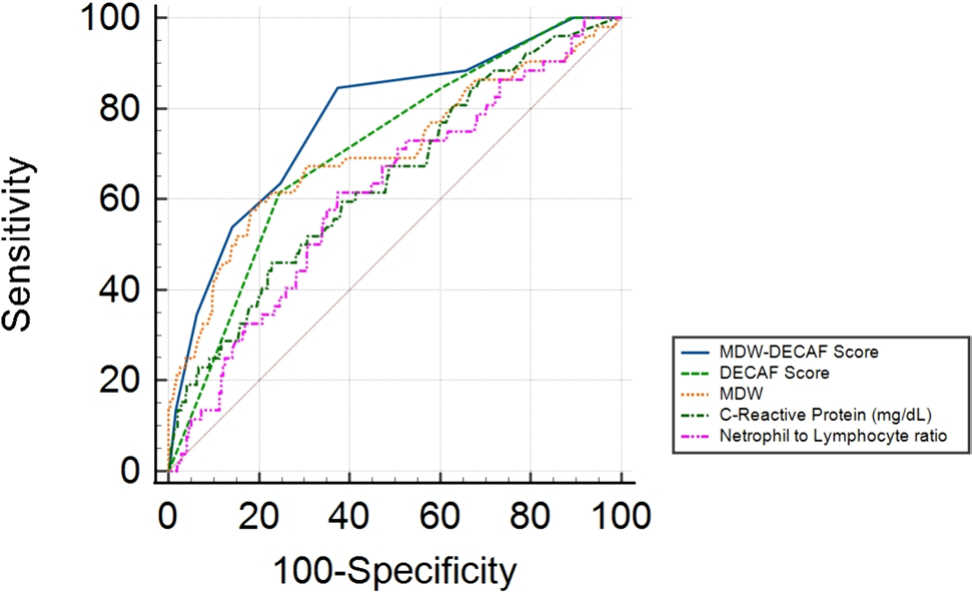
Receiver operator characteristic curve showing the discrimination ability of MDW, C-reactive protein, neutrophil to lymphocyte ratio, the DECAF score and the MDW–DECAF score for in-hospital mortality or ICU admission. *MDW* monocyte distribution width, *DECAF *dyspnea, eosinopenia, consolidation, acidemia and atrial fibrillation score

According to our findings, we created a new tool, the MDW–DECAF score, based on the DECAF score and including the same variables as DECAF and the MDW, with a relative weight assigned according to the regression coefficient for the composite end point (3 points). In ROC analysis (Fig. 3), the MDW–DECAF score’s AUC for differentiating patients who died or were admitted to the ICU from the rest of the patients (AUC 0.777 95% IC 0.708–0.845, *p* < 0.001) had the best diagnostic power and was followed by the DECAF score (AUC 0.710 95% IC 0.639–0.782, *p* < 0.001) and MDW (AUC 0.705 95% IC 0.618–0.791, *p* < 0.001) (Fig. 3, Supplementary file 1). Youden’s index for MDW was > 21.7, with a sensitivity of 57.69 and a specificity of 81.95. Youden’s index for the MDW–DECAF score was > 2, with a sensitivity of 84.62 and a specificity of 63.01. MDW–DECAF had a statistically significantly higher AUC than the DECAF score (*p* = 0.023), MDW (*p* = 0.026), C-RP (*p* = 0.002) and neutrophil to lymphocyte ratio. No statistically significant differences were found among the AUC values of the remaining variables.

## Discussion

This study provides the first demonstration that MDW is associated with the severity of severe ECOPD and can be used as a predictor of mortality and ICU admission. Moreover, it introduces the new MDW–DECAF score.

Patients with high MDW, as reported by previous studies in patients with systemic inflammatory response syndrome [28, 29] or COVID-19 [30], had elevated C-RP, DECAF scores and neutrophil to lymphocyte ratios, and lower blood eosinophils; these prognostic factors are well known to be associated with both ECOPD inflammatory response and severity [10, 11, 31–33]. We also evaluated variables associated with higher MDW values in a multivariate logistic regression analysis, which indicated that C-RP, mortality and ICU admission were associated with high MDW values. Our findings indicate the importance of inflammation in MDW levels, as discussed in other clinical contexts, such as sepsis [16, 19, 20, 24, 28, 29], COVID-19 [22–24], influenza [34] and complicated diverticulitis [35].

Our findings provide the first evidence that the MDW values according to blood tests performed after emergency department arrival predict death and ICU admission for ECOPD. This novel finding has not previously been described in the context of COPD, but has been reported in other settings, such as COVID-19 [23] and sepsis [28]. Furthermore, our results were obtained by using Cox regression analysis considering the time to the event, whereas other studies have evaluated ECOPD prognosis by using only logistic regression analysis [10, 11]. Additionally, the cutoff point of the test was determined on the basis of previous studies [28–30]. This was similar to Youden’s index in our study.

The AUC of MDW for predicting the prognosis of severe ECOPD in terms of mortality or ICU admission was comparable to that of other well-known inflammatory markers of COPD, such as C-RP [9] or the neutrophil to lymphocyte ratio [11]. MDW is biomarker that can be routinely measured rapidly, easily and inexpensively in emergency departments. Further studies are necessary to evaluate the limitations and benefits of each biomarker in COPD. The DECAF score [10] is a well-known risk stratification tool for patients with severe ECOPD, but its AUC can be improved by using novel biomarkers. Because MDW is a different predictor from the DECAF score for mortality or ICU admission, we created a new score, the MDW–DECAF score, which had a better AUC than the other biomarkers or the DECAF score alone. Further studies are required to evaluate the potential roles of these scores in assessing specific etiologies of ECOPD or inflammatory conditions.

This study has several strengths. First, it is a novel study reporting the first evaluation of MDW in ECOPD in real-world circumstances. Second, this was a multicenter study in patients from three hospitals. Finally, the patients included in this study were carefully selected and well characterized, and patients with diseases or therapies that might have influenced the results were excluded.

However, our study also has several limitations. First, this was a retrospective study and therefore was subject to a risk of information bias. Although all routine blood tests were performed minutes after arrival at the emergency department, the retrospective nature of this study could imply different timing for analysis from whole blood venous sample collection. Furthermore, our results cannot be extrapolated to all patients with COPD since we excluded patients with hematological and nutritional conditions. Our findings should be replicated in other settings, in a larger number of patients, with standardized therapy and multiple samples, to evaluate the time course of the responses of MDW. Future specifically designed prospective studies should be performed to evaluate the utility of MDW and externally validate MDW–DECAF.

## Conclusion

Our study provides the first evidence that MDW is associated with ECOPD severity and predicts mortality and ICU admission with a diagnostic accuracy similar to that of DECAF and C-RP. Furthermore, on the basis of our results, we created a new tool, the MDW–DECAF score, which has better diagnostic accuracy than the DECAF score in identifying mortality or ICU admission.

## Supplementary Information

Below is the link to the electronic supplementary material.

Supplementary file1 (DOCX 14 KB)

## Data Availability

The datasets used and/or analysed during the current study available from the corresponding author on reasonable request.

## References

[1] GBD Chronic Respiratory Disease Collaborators (2020) Prevalence and attributable health burden of chronic respiratory diseases, 1990–2017: a systematic analysis for the global burden of disease study 2017. Lancet Respir Med 8:585–596 32526187 10.1016/S2213-2600(20)30105-3 PMC7284317

[2] Soler-Cataluña JJ, Martínez-García MA, Román Sánchez P, Salcedo E, Navarro M, Ochando R (2005) Severe acute exacerbations, and mortality in patients with chronic obstructive pulmonary disease. Thorax 60:925–931 16055622 10.1136/thx.2005.040527 PMC1747235

[3] Dransfield MT, Kunisaki KM, Strand MJ, Anzueto A, Bhatt SP, Bowler RP, Criner GJ, Curtis JL, Hanania NA, Nath H, Putcha N, Roark SE, Wan ES, Washko GR, Wells JM, Wendt CH, Make BJ, COPDGene Investigators (2017) Acute exacerbations and lung function loss in smokers with and without chronic obstructive pulmonary disease. Am J Respir Crit Care Med 195:324–330 27556408 10.1164/rccm.201605-1014OC PMC5328181

[4] Spencer S, Jones PW, GLOBE Study Group (2003) Time course of recovery of health status following an infective exacerbation of chronic bronchitis. Thorax 58:589–593 12832673 10.1136/thorax.58.7.589 PMC1746751

[5] Suissa S, Dell’Aniello S, Ernst P (2012) Long-term natural history of chronic obstructive pulmonary disease: severe exacerbations and mortality. Thorax 67:957–963 22684094 10.1136/thoraxjnl-2011-201518 PMC3505864

[6] Jacobs DM, Noyes K, Zhao J, Gibson W, Murphy TF, Sethi S, Ochs-Balcom HM (2018) Early hospital readmissions after an acute exacerbation of chronic obstructive pulmonary disease in the nationwide readmissions database. Ann Am Thorac Soc 15:837–845 29611719 10.1513/AnnalsATS.201712-913OC PMC6207114

[7] Connors AF, Dawson NV, Thomas C et al (1996) Outcomes following acute exacerbation of severe chronic obstructive lung disease. The SUPPORT investigators (study to understand prognoses and preferences for outcomes and risks of treatments). Am J Respir Crit Care 154:959–967 10.1164/ajrccm.154.4.8887592 8887592

[8] Gunen H, Hacievliyagil SS, Kosar F et al (2005) Factors affecting survival of hospitalised patients with COPD. Eur Respir J 26:234–241 16055870 10.1183/09031936.05.00024804

[9] GOLD (2023) Global initiative for chronic obstructive lung disease. GOLD, Fontana 10.2174/0118743064279064231227070344 PMC11037508 38660684

[10] Steer J, Gibson J, Bourke SC (2012) The DECAF score: predicting hospital mortality in exacerbations of chronic obstructive pulmonary disease. Thorax 67:970–976 22895999 10.1136/thoraxjnl-2012-202103

[11] Lu FY, Chen R, Li N, Sun XW, Zhou M, Li QY, Guo Y (2021) Neutrophil-to-lymphocyte ratio predicts clinical outcome of severe acute exacerbation of COPD in frequent exacerbators. Int J Chron Obstruct Pulmon Dis 17(16):341–349 10.2147/COPD.S290422 PMC7901567 33633446

[12] Yang J, Qiao M, Li Y, Hu G, Song C, Xue L, Bai H, Yang J, Yang X (2018) Expansion of a population of large monocytes (atypical monocytes) in peripheral blood of patients with acute exacerbations of chronic obstructive pulmonary diseases. Mediators Inflamm 2018:9031452 29887758 10.1155/2018/9031452 PMC5985121

[13] Dilmoula A, Kassengera Z, Turkan H (2011) Volume, conductivity, and scatter properties of leukocytes (VCS technology) in detecting sepsis in critically ill adult patients. Blood 118:4729 10.1182/blood.V118.21.4729.4729

[14] Celik IH, Demirel G, Askoy HT (2012) Automated determination of neutrophil VCS parameters in diagnosis and treatment efficacy of neonatal sepsis. Pediatr Res 71:121–125 22289860 10.1038/pr.2011.16

[15] Bhargava M, Saluja S, Sindhuri U, Saraf A, Sharma P (2014) Elevated mean neutrophil volume + CRP is a highly sensitive and specific predictor of neonatal sepsis. Int J Lab Hematol 36:e11–e14 23795566 10.1111/ijlh.12120

[16] Lee AJ, Kim SG (2013) Mean cell volumes of neutrophils and monocytes are promising markers of sepsis in elderly patients. Blood Res 48:193–197 24086939 10.5045/br.2013.48.3.193 PMC3786279

[17] Chaves F, Tierno B, Xu D (2005) Quantitative determination of neutrophil VCS parameters by the coulter automated hematology analyzer: new and reliable indicators for acute bacterial infection. Am J Clin Pathol 124:440–444 16191513 10.1309/LLF75W0FWQQ8TCC5

[18] Chaves F, Tierno B, Xu D (2006) Neutrophil volume distribution width: a new automated hematologic parameter for acute infection. Arch Pathol Lab Med 130:378–380 16519568 10.5858/2006-130-378-NVDWAN

[19] Mardi D, Fwity B, Lobmann R, Ambrosch A (2010) Mean cell volume of neutrophils and monocytes compared with C-reactive protein, interleukin-6, and white blood cell count for prediction of sepsis and nonsystemic bacterial infections. Int J Lab Hematol 32:410–418 19919621 10.1111/j.1751-553X.2009.01202.x

[20] Crouser ED, Parrillo JE, Seymour C, Angus DC, Bicking K, Tejidor L, Magari R, Careaga D, Williams J, Closser DR, Samoszuk M, Herren L, Robart E, Chaves F (2017) Improved early detection of sepsis in the ED with a novel monocyte distribution width biomarker. Chest 152:518–526 28625579 10.1016/j.chest.2017.05.039 PMC6026271

[21] Lippi G, Sanchis-Gomar F, Henry BM (2021) Pooled analysis of monocyte distribution width in subjects with SARS-CoV-2 infection. Int J Lab Hematol 43:O161–O163 33554458 10.1111/ijlh.13482 PMC8013932

[22] Lin HA, Lin SF, Chang HW, Lee YJ, Chen RJ, Hou SK (2020) Clinical impact of monocyte distribution width and neutrophil-to-lymphocyte ratio for distinguishing COVID-19 and influenza from other upper respiratory tract infections: a pilot study. PLoS ONE 15:e0241262 33137167 10.1371/journal.pone.0241262 PMC7605646

[23] Lorubbio M, Tacconi D, Iannelli G, Feri M, Scala R, Montemerani S, Mandò M, Ognibene A (2022) The role of monocyte distribution width (MDW) in the prognosis and monitoring of COVID-19 patients. Clin Biochem 103:29–31 35182522 10.1016/j.clinbiochem.2022.02.007 PMC8848545

[24] Alsuwaidi L, Al Heialy S, Shaikh N, Al Najjar F, Seliem R, Han A, Hachim M (2022) Monocyte distribution width as a novel sepsis indicator in COVID-19 patients. BMC Infect Dis 22:27 34983404 10.1186/s12879-021-07016-4 PMC8724663

[25] Miravitlles M, Calle M, Molina J, Almagro P, Gómez JT, Trigueros JA et al (2022) Spanish COPD guidelines (GesEPOC) 2021: updated pharmacological treatment of stable COPD. Arch Bronconeumol 58:69–81 33840553 10.1016/j.arbres.2021.03.005

[26] García-Río F, Calle M, Burgos F, Casan P, Del Campo F, Galdiz JB, Giner J, González-Mangado N, Ortega F, Puente ML (2013) Spanish society of pulmonology and thoracic surgery (SEPAR) spirometry. Arch Bronconeumol 49:388–401 23726118 10.1016/j.arbres.2013.04.001

[27] Charlson ME, Pompei P, Ales KL, MacKenzie CR (1987) A new method of classifying prognostic comorbidity in longitudinal studies: development and validation. J Chronic Dis 40:373–383 3558716 10.1016/0021-9681(87)90171-8

[28] Wu J, Li L, Luo J (2022) Diagnostic, and prognostic value of monocyte distribution width in sepsis. J Inflamm Res 15:4107–4117 35898818 10.2147/JIR.S372666 PMC9309295

[29] Hausfater P, Robert Boter N, Morales Indiano C, Cancella de Abreu M, Marin AM, Pernet J et al (2021) Monocyte distribution width (MDW) performance as an early sepsis indicator in the emergency department: comparison with CRP and procalcitonin in a multicenter international European prospective study. Crit Care 25:227 34193208 10.1186/s13054-021-03622-5 PMC8247285

[30] Wakamatsu K, Nagasawa Z, Katsuki K, Kumazoe H, Yasuda M, Kawamoto S, Kawamura A, Ueno T, Kiyotani R, Fukui I, Maki S, Nagata N, Kawasaki M, Yamada H (2023) Retrospective study on the efficacy of monocyte distribution width (MDW) as a screening test for COVID-19. Eur J Med Res 28:136 36973757 10.1186/s40001-023-01086-7 PMC10040926

[31] Stolz D, Christ-Crain M, Morgenthaler NG, Leuppi J, Miedinger D, Bingisser R, Müller C, Struck J, Müller B, Tamm M (2007) Copeptin, C-reactive protein, and procalcitonin as prognostic biomarkers in acute exacerbation of COPD. Chest 131:1058–1067 17426210 10.1378/chest.06-2336

[32] Antonescu-Turcu AL, Tomic R (2009) C-reactive protein and copeptin: prognostic predictors in chronic obstructive pulmonary disease exacerbations. Curr Opin Pulm Med 15:120–125 19532026 10.1097/MCP.0b013e3283218603

[33] de Torres JP, Cordoba-Lanus E, López-Aguilar C, Muros de Fuentes M, Montejo de Garcini A, Aguirre-Jaime A, Celli BR, Casanova C (2006) C-reactive protein levels and clinically important predictive outcomes in stable COPD patients. Eur Respir J 2006(27):902–907 10.1183/09031936.06.00109605 16455829

[34] Badaki-Makun O, Levin S, Debraine A, Hernried B, Malinovska A, Smith A, Toerper M, Fenstermacher KZJ, Cottle T, Latallo M, Rothman RE, Hinson JS (2022) Monocyte distribution width as a pragmatic screen for SARS-CoV-2 or influenza infection. Sci Rep 12:21528 36513693 10.1038/s41598-022-24978-w PMC9745720

[35] Chang CY, Hsu TY, He GY, Shih HM, Wu SH, Huang FW, Chen PC, Tsai WC (2023) Utility of monocyte distribution width in the differential diagnosis between simple and complicated diverticulitis: a retrospective cohort study. BMC Gastroenterol 23:96 36977993 10.1186/s12876-023-02736-0 PMC10047462

