# Supplementary material for: Monocyte distribution width (MDW) and DECAF: two simple tools to determine the prognosis of severe COPD exacerbation
Source: Intern Emerg Med. 2024 May 9;19(6):1567–75. doi: 10.1007/s11739-024-03632-5 (PMC11405499; doi:10.1007/s11739-024-03632-5)
Supplement: Supplementary file 1 — Supplementary file1 (DOCX 14 KB) [file 11739_2024_3632_MOESM1_ESM.docx]

**Supplementary file 1:** Patients who died or were admitted in UCI vs. the remaining patients.

|  | **AUC** | **OR (95% CI)** | **p** |
| --- | --- | --- | --- |
| **MDW (units)** | **0.705** | **0.618-0.791** | **<0.001** |
| **C-RP (mg/dL)** | **0.643** | **0.562-0.723** | **0.001** |
| **Neutrophils/Lymphocytes** | **0.614** | **0.533-0.694** | **0.008** |
| **DECAF Score** | **0.710** | **0.639-0.782** | **<0.001** |
| **MDW-DECAF Score** | **0.777** | **0.708-0.845** | **<0.001** |

AUC= area under curve, MDW= monocyte distribution width, C-RP= C-reactive protein, DECAF= dyspnea, eosinopenia, consolidation, acidemia and atrial fibrillation score, MDW-DECAF= MDW, dyspnea, eosinopenia, consolidation, acidemia and atrial fibrillation score. Bold font indicates statistical significance.
